# Observing and assessing role play in early childhood: evaluation of the Play Matrix tool

**DOI:** 10.3389/fpsyg.2025.1614161

**Published:** 2025-12-02

**Authors:** Nikolay Veraksa, Nikolay Veresov, Vera Sukhikh, Valeriya Plotnikova

**Affiliations:** 1 Department of Educational Psychology and Pedagogy, Faculty of Psychology, Lomonosov Moscow State University, Moscow, Russia; 2 Laboratory for Child Psychology and Digital Socialization, Federal Scientific Centre for Psychological and Multidisciplinary Research, Moscow, Russia; 3 Faculty of Education, Monash University, Melbourne, VIC, Australia

**Keywords:** free play, adult-facilitated play, guided play, role-play assessment, self-regulation, effortful control, early childhood development

## Abstract

This study evaluates the Play Matrix, a novel observational tool designed to assess role-play in early childhood within the cultural-historical framework. The Play Matrix comprises 22 behavioral indicators across actions, speech, and emotional expressions. We examined its reliability and validity by analyzing video recordings of 42 children aged 5–6 years in both free play and adult-facilitated play settings. High inter-rater reliability was observed for most indicators, and significant differences in play behaviors between children with high and low levels of self-regulation were detected. Children with lower self-regulation exhibited more impulsive actions, expressive gestures, and involvement in emotionally rich group actions. Additionally, to validate the Play Matrix as a tool for analyzing the nuances of play situations, play behaviors in different play situations was compared. It was noted that children in free play show more unscripted character actions, speech in the play context, and more often regulate the behavior of other children. The Play Matrix offers a comprehensive mechanism for capturing the developmental potential of play, providing valuable insights into early childhood development.

## Introduction

Despite the extensive body of research on children’s play, interest in this subject continues to intensify (Yogman et al., 2018; Prins et al., 2022). This sustained interest is attributable to the fundamental role of play in the developmental trajectory of preschool children (Elkonin, 2005; Yogman et al., 2018). Empirical evidence substantiates the influence of play on various dimensions of a child’s psychological development (Yogman et al., 2018; Ginsburg et al., 2007). Play serves as a principal modality in psychotherapeutic and corrective interventions with children (Koukourikos et al., 2021; Ritvo, 2016), and is employed as a diagnostic tool for assessing child development and psychological wellbeing (Salcuni et al., 2017; Ritvo, 2016). Globally, educational institutions are integrating play-based curricula (Pyle et al., 2017), and effective strategies for fostering children’s play by educators are being investigated and implemented (Ndlovu et al., 2023; Parker et al., 2022).

Nevertheless, there is a paucity of instruments designed for the observation and assessment of play and what is particularly missing in current approaches is a tool that allows in-the-moment observation of play—capturing it as it unfolds. This scarcity is primarily due to the inherent complexity of play. Play encompasses a diverse array of types and a multifaceted structure. For example, play involving imaginary situations evolves and transforms throughout the preschool years (Elkonin, 2005; Piaget, 1952). The deficit of observational tools is particularly pronounced in scientific research, where a high degree of differentiation is required to capture and operationalize the various aspects and forms of pretend play. The Play Matrix was developed within the cultural-historical approach as a tool to address a broad spectrum of research objectives (Veraksa et al., 2022). This is achieved through its extensive array of indicators and their high degree of differentiation. This study evaluates the reliability and validity of the Play Matrix showing its high potential for studying play behaviors in various forms of play situations. The Play Matrix provide the analysis of the play situation from the child’s perspective and shows the degree of the child effortful control and involvement in the process.

## Conceptual framework and development of the Play Matrix

The cultural-historical approach is one of the most prevalent theoretical frameworks in play research. This approach allows researchers to clearly define what play is, identify its key components, and delineate the stages and levels of its development. Furthermore, it articulates the mechanisms and conditions through which play can exert a long-term developmental impact on children who engage in it (Fleer, 2022; Sukhikh et al., 2023; Veraksa et al., 2020, 2023; Vygotsky, 2005).

Within the framework of the cultural-historical approach, play creates a zone of proximal development for the child, primarily – for effortful control (Vygotsky, 2016; Hakkarainen and Bredikyte, 2008; Zakharova and Machinskaya, 2023). This developmental stage typically emerges around the age of three with the initial manifestations of pretense. According to the cultural-historical perspective, all elements of play—roles, rules, narratives, play actions, and play props — have sociocultural nature. The child’s behavior during play elucidates the development of higher psychological functions as intrinsic psychological resources, which the child utilizes to plan, monitor, and regulate their activities, thereby actualizing culturally normative behaviors (Vygotsky, 2005).

An examination of assessment tools for play reveals that this very process of child effortful control remains challenging to identify and further analyze. Typically, play is employed as a tool or context for evaluating the child’s social adaptation, affective states, or cognitive domains. For instance, Penn Interactive Peer Play Scale (PIPPS) (Fantuzzo et al., 1995) is a teacher-rating instrument to observe children’s behavior and assess it via Likert scale. It describes positive and negative play behaviors on three dimensions (play interaction, play disruption, play disconnection) in order to identify children who need help and guide intervention efforts. In Test of Pretend Play (ToPP) (Lewis and Boucher, 1997) a child is provided with instructions for symbolic play to test their abilities for substituting one object for another, reference to an absent object as if it was present and attributing an imaginary property to an object. Additionally, ToPP examines how well a child can incorporate symbolic play actions into a coherent sequence. The information obtained through the methodology serves as the basis for assessing the child’s level of cognitive development. The Child-Initiated Pretend Play Assessment (ChIPPA) (Stagnitti, 2007) was developed for a clinical setting. It is used to evaluate preschoolers’ ability to self-initiate make-believe free play using a conventional–imaginative and unstructured set of toys. It assesses complexity, elaborateness and organization of child’s play using three items of observed behavior: percentage of elaborate pretend play actions, number of object substitutions, and number of imitated actions. The method allows to detect any issues a child may exhibit in play behavior (Uren and Stagnitti, 2009). The Play Observational Scale (POS) (Rubin, 2001) offers description of play and non-play behaviors used to analyze and categorize children’s predominant activity. It just detects the type of play (functional play, constructive play, dramatic play, and games-with-rules) and the nature of its social form – whether it is solitary, parallel or group play. Thus, these tools are designed for various practical purposes, focus on some aspects of the play and do not provide a holistic view of the level of role-play as a complex activity.

The assessment tools for play developed within the framework of the cultural-historical approach are the most comprehensive in terms of understanding the structure of play. Since these tools, unlike those listed above, are developed within the same theoretical paradigm, they are built in a similar way and their brief comparative description is provided in a Table 1. All of the three tools are designed for the observation of collective socio-dramatic play, which is the most advanced form of play involving an imaginary situation. However, these tools do not provide the evaluation of the play situation from the child’s perspective: does the child successfully navigates actions in both imaginary and real worlds, what is the child’s behavior in the role (whether they introduce novel elements or merely follow the plot formally, whether they remain engaged in the play throughout its duration or become distracted, etc.), and the degree of the child’s emotional involvement in the process. These aspects of play are crucial as they significantly determine its developmental potential (Veresov, 2019; Ryabkova and Sheina, 2023, Yudina, 2022).

**TABLE 1:** Comparative description of play assessment tools developed within the framework of the cultural-historical approach.

Measure	Indicators	Assessment setting
Smilansky Scale for the Evaluation of Dramatic and Socio-Dramatic Play (SSEDSP) (Smilansky and Shefatya, 1990)	1. Role play. 2. Make-believe with objects. 3. Make-believe with actions and situations. 4. Persistence in role play. 5. Interaction. 6. Verbal communication.	Observation of free play in natural settings. The play environment should include materials related to household activities, a hospital, a store, open-ended materials, dress-up clothes, and a set of tools. Children’s play is measured both in individual and group play.
Mature Make-Believe Play Observational Instrument (MPOT) (Germeroth et al., 2019)	1. Child-created props. 2. Metaplay. 3. Play interactions. 4. Role playing. 5. Role speech and communication.	Observation of free play in classroom settings.
The level of play development assessment (Smirnova et al., 2018)	1. Level of substitution. 2. Interaction among children (organizing the play and within-play interaction). 3. Play idea (elaboration, stability, etc.).	In a playroom a small group of 3–5 children is provided with open-ended, multifunctional materials. Then children engage in a free play. This setting allows for the assessment of play in specially created conditions, free from adult suggestions or predefined images embodied in toys.

To address this gap, the Play Matrix was developed, comprising 22 behavioral indicators categorized into three domains: actions, speech actions, and emotional expressions. Each domain, along with the conceptual pillars that guided the development of this tool, was comprehensively detailed in a prior publication (Veraksa et al., 2022). The indicators of the Play Matrix were effectively utilized for the qualitative analysis of video recordings of play sessions conducted during a training experiment (Sukhikh et al., 2022). Based on this empirical application, the tool went through refinement. The definitions and descriptions of the indicators were articulated with greater precision and clarity. Additionally, options for combining indicators into broader units were formulated, which can significantly simplify the coding process when necessary. A criterion of uncertainty was also introduced—this category is intended to encompass all manifestations that, due to insufficient information, cannot be unequivocally classified based on objective observation without the interpretative judgments of the observer. Instructions for observers were meticulously prepared, and training sessions were conducted.

These steps facilitated the preparation of the tool for broader application to rigorously assess its reliability and validity. To this end, we examined (1) inter-rater reliability; (2) correlations between child play indicators (as indexed by the Play Matrix) and relevant child outcomes, such as self-regulation; and (3) the differences in children’s play behaviors (as indexed by the Play Matrix) in different play situations — free play versus play with active adult participation. The key hypothesis was the assumption that the Play Matrix captures the differences in the play behavior for the children with low and high levels of effortful control development.

## Materials and methods

### Procedure

The study included 42 children (57% male), aged 5–6 years (*M* = 70, SD = 3.72). All children with typical development attended kindergartens in the city of Moscow. Informed consent for participation and video recording was obtained from the parents of all participants. The study was approved by the Commission on Ethics of Scientific Research of the Federal Scientific Center for Psychological and Interdisciplinary Research (No. 3 dated 01/31/2024). Each child underwent a standardized individual testing session, followed by engagement in role-play with peers in groups of 5–8 participants. The role-play sessions were recorded from two points of the room. The children were allowed to utilize the entire room, including furniture, and were provided with open-ended multifunctional materials to serve as props and substitute objects. The average duration of the play sessions was 15–20 min. A subsample of 25 children participated in role-play activities facilitated by an adult. Member of the research team provided plot suggestions on the basis of a book which was read and discussed with a group of children before the play sessions, assisted with role distribution, props creation and had a role in play, so could interact and facilitate the play as a character within the plot. 17 children were involved in a play free from any adult intervention. The total sample size was 42 children. A total of 10 of them were chosen to be evaluated by three coders, with five participating in a free play and five – in adult-facilitated play. However, due to the technical difficulties, one of the three coders evaluated only eight children, and two others – all of 10. For each of 42 children the same segment of video recording was assessed – 15 min from the start of the play, not including the preparatory stage (630 min - total duration of the material coded and analyzed).

The coders from the research team with the degree in psychology and extensive experience in research included preschool children, were trained by co-authors of the Play Matrix. They independently transcribed the recordings and coded every recorded behavioral manifestation according to the Play Matrix indicators.

### Measures

#### Self-regulation measures

Effortful control was assessed as the level of development of the three components of executive functions (EF) (Miyake et al., 2000) by NEPSY-II subtests (Almazova et al., 2019; Korkman et al., 2007). Visual working memory was measured by “Memory for Designs” subtest that required remembering the pictures and their location. Verbal working memory was assessed by “Sentence Repetition” subtest, where children were to repeat the sentences that gradually became more complex, both grammatically and lexically. “Statue” subtest assessed physical inhibitory control. Hot self-regulation was measured by “Walk-a-Line-Slowly” test (Maccoby et al., 1965), where children were required to walk on the line as slowly as possible, there and back. Meanwhile, they were able to see the toys that could be played with after completing the task. Cognitive flexibility was assessed by “The Dimensional Change Card Sort” test which requires to sort the cards in accordance with changing rules (Almazova et al., 2019; Veraksa et al., 2023; Zelazo, 2006).

#### The Play Matrix indicators

To evaluate the video recordings, nearly all indicators from the original Play Matrix were utilized, with some indicators being consolidated into larger units resulting in a total of 12 indicators. The units — “inconsistencies,” “speech in the play context,” “character speech,” “expressive gestures and emotional responses” — were based on indicators that share common nature and interpretation (Table 2). The same behavior manifestation could be attributed to several indicators.

**TABLE 2:** Description of the Play Matrix indicators.

Actions		
Indicator		Definition, examples
1.1	Impulsive actions	Sudden behavior out of the play context. The child can’t wait still, shouts, jumps simply to stay active.
1.2	Field actions	Behavior focused on environmental stimuli. The child fixates on a toy or other random object, gets distracted from the play.
1.3	Character actions	Actions that are typical for a character. The Knight “rides” into the castle at a normal pace or slightly bouncing; swings the “sword” or brings a bowl to his mouth, as if there was food in it. The child does not develop the character.
1.4	Unscripted character actions	Unique actions or character props originally brought by the child and consistent with the general plot. Any expressions of the child’s initiative and individuality in role-playing. When faced with the problem of a kidnapped Princess, the child takes the initiative and throws a “net” on the Dragon to capture it.
1.5	Inconsistency	A unit for all cases of deviation from the plot and falling out of character: *actions in other play contexts, voluntary actions outside the play context, and spontaneous actions not aligned with the general plot* from the original Play Matrix. It is noteworthy that, unlike impulsive and field actions, these actions are meaningful to the child and relate to the play, yet they do not currently align with it in various ways.
**Emotional manifestations**		
2.1	Expressive gestures and emotional responses	An emotional reaction to a situation within the play which appears in gestures and facial expressions. The child laughs and/or is surprised in response to what is happening in the play; looks at another child with resentment, stamps a foot. Exclamations in the form of interjections, words, or entire phrases: “This is a sword!” Any vivid emotional expressions by the child—verbal or non-verbal. Gestures, role-playing actions, and statements with pronounced emotional tone.
2.2	Emotionally rich group actions	Several children are involved in a play episode at once and express themselves emotionally with loud voices/gestures/facial expressions/exclamations and so on. A battle involving several characters, active discussion in a «feast».
**Verbal manifestations**		
**Indicator**		**Examples**
3.1	Character speech (character’s lines), naming their role, their in-game actions	Speech belonging to a child’s character as part of the plot development. One manifestation is considered to be a complete segment in terms of meaning – a word, phrase or even several sentences.
3.2	Regulating the behavior of other children	A child tells other children what to do and how: «Go there and wait».
3.3	Discussing the development of the play with playmates	Children introduce their suggestions on the play development and evaluate the suggestions of others.
3.4	Speech in the play context	A unit for all cases of the play related speech manifestations: *comments, judgments related to the play, meta-reflective speech* from the original Play Matrix.
**Indetermined**		
	Indeterminate actions, statements, emotional manifestations	Something that cannot be classified into any category without speculation. There is not enough information to attribute the manifestation to a specific indicator.

### Data analysis

The frequency of indicators was considered, due to the study aim the Play Matrix intended to be fully completed for each observation. The design of the Play Matrix does not suggest the overall assessment, each category was analyzed. Statistical analysis of the collected data included inter-rater reliability using the Intraclass Correlation Coefficient (ICC), specifically ICC(C,3) for consistency, criterion validity using cluster analysis and the Kruskal-Wallis test (Kruskal and Wallis, 1952), and correlation analysis using Spearman’s correlation. The validity of the Play Matrix was also assessed by comparing play behaviors in different play situations using the Kruskal-Wallis test.

## Results

### Inter-rater reliability

The reliability analysis of the assessments by three coders was conducted for two parameters: (1) the number of indicators identified by each expert using the Play Matrix, and (2) the child’s play profile, which encompasses the frequency of manifestations of each identified indicator. The inter-rater reliability for the 12 behavioral indicators and the total number of identified indicators, as assessed by the three coders, was evaluated using the Intraclass Correlation Coefficient (ICC), specifically ICC(C,3) for consistency. This form of ICC measures reliability based on the average assessments of the three coders. The results are summarized in a Table 3. The missing data were not included in the ICC calculations, which were performed using only the available data.

**TABLE 3:** Intraclass Correlation Coefficient (ICC) results for coders assessments of behavioral indicators in child’s play.

Parameter	ICC(C,3)	*F*-value	*P*-value	95% confidence interval
Impulsive actions	0.774**	4.43	0.0087	0.236 < ICC < 0.951
Field actions	0.89**	9.07	0.0003	0.627 < ICC < 0.976
Character actions	0.868**	7.55	0.0007	0.553 < ICC < 0.971
Unscripted character actions	0.727**	3.66	0.0185	0.077 < ICC < 0.941
Inconsistency	0.767**	4.3	0.0098	0.214 < ICC < 0.949
Expressive gestures and emotional responses	0.781**	4.58	0.0075	0.261 < ICC < 0.952
Emotionally rich group actions	0.969***	31.8	1.49e-07	0.894 < ICC < 0.993
Character speech	0.838**	6.16	0.0020	0.451 < ICC < 0.965
Regulating the behavior of other children	0.651*	2.86	0.0446	−0.18 < ICC < 0.924
Discussing the development of the play with playmates	Zero variability			
Speech in the play context	0.202	1.25	0.34	−1.698 < ICC < 0.826
Indeterminate actions, statements, emotional manifestations	0.652*	2.87	0.0440	−0.176 < ICC < 0.924
Total number of categories	0.825**	5.73	0.0028	0.41 < ICC < 0.962

*, good consistency;

**, high consistency;

***, very high consistency.

Most indicators show good to excellent consistency among coders, with statistically significant F-values indicating reliable measurements. For the “Discussing the development of the play with playmates” indicator, all ratings provided by the raters were zero, indicating that no suggestions-related actions were observed for any of the subjects. This resulted in zero variability among the ratings, which means the raters were in complete agreement that no actions related to “Discussing the development of the play with playmates” were performed by the subjects. Consequently, the ICC calculation for this parameter could not be performed, and it is reported as having zero variability. The “Speech in the play context” parameter showed low consistency and high variability, suggesting unreliable ratings for this indicator.

### Criterion validity: the Play Matrix indicators and effortful control

To validate the Play Matrix as a tool for assessing the level of effortful control during the role-play, behavioral differences among children with varying levels of EF development (as determined by test results) were analyzed. Employing cluster analysis (K-means clustering), the children were categorized into two subgroups: those with low (*N* = 20) and high (*N* = 22) levels of EF development. It was hypothesized that the observed differences would be associated with indicators directly related to effortful control in play: impulsive actions, field actions, inconsistency, emotionally rich group actions, expressive gestures and emotional responses and regulating the behavior of other children.

A Kruskal-Wallis test was conducted to examine the differences between children with high and low levels of self-regulation across the Play Matrix indicators. The analysis revealed significant differences in the number of impulsive actions, χ^2^(1, *N* = 42) = 10.4, *p* = 0.001, η^2^ = 0.253, expressive gestures and emotional responses χ^2^(1, *N* = 42) = 4.31, *p* = 0.038, η^2^ = 0.1052, involvement in emotionally rich group actions, χ^2^(1, *N* = 42) = 4.57, *p* = 0.033, η^2^ = 0.111. Children with low levels of self-regulation exhibited significantly more impulsive actions, expressive reactions during play, and involvement in group emotionally charged activities compared to children with high levels of self-regulation (see Table 4). No other significant differences were detected.

**TABLE 4:** Descriptive statistics and results of non-parametric one-way analysis of variance for children with different levels of executive functions (EF).

Parameter	Mean		SD		Min		Max		χ^2^, Kruskal-Wallis test	df	*P*-value	Effect size, e^2^
	Low EF level	High EF level	Low EF level	High EF level	Low EF level	High EF level	Low EF level	High EF level				
Impulsive actions	5.45	2.14	5.21	3.76	0.00	0.00	20.0	12.0	10.399	1	0.001*	0.25363
Field actions	4.00	1.95	4.68	2.77	0.00	0.00	18.0	9.00	2.498	1	0.114	0.06092
Character actions	7.20	6.73	8.16	4.04	0.00	0.00	33.0	14.0	0.812	1	0.368	0.01980
Unscripted character actions	0.250	0.0909	1.12	0.294	0.00	0.00	5.00	1.00	0.204	1	0.652	0.00497
Inconsistency	1.20	0.500	1.85	1.54	0.00	0.00	7.00	7.00	3.586	1	0.058	0.08747
Expressive gestures and emotional responses, emotional exclamations	4.20	2.77	3.72	3.32	1.00	0.00	16.0	11.0	4.312	1	0.038*	0.10517
Emotionally rich group actions	4.40	2.50	2.72	2.56	0.00	0.00	8.00	8.00	4.568	1	0.033*	0.11141
Character speech	3.45	1.91	5.24	2.04	0.00	0.00	22.0	7.00	0.522	1	0.470	0.01274
Regulating the behavior of other children	0.200	0.182	0.523	0.588	0.00	0.00	2.00	2.00	0.243	1	0.622	0.00592
Suggestions on how to proceed the play	0.100	0.0455	0.308	0.213	0.00	0.00	1.00	1.00	0.459	1	0.498	0.01119
Speech in the play context	2.35	1.82	2.30	2.94	0.00	0.00	8.00	9.00	2.372	1	0.124	0.05784

*Significant results (*p*-value < 0.05).

### Criterion validity: the Play Matrix indicators and the play situation

To validate the Play Matrix as a tool for analyzing the nuances of play situations, play behaviors in different play situations was compared. Play situations varied in the degree of freedom for children to exhibit initiative and individuality. In adult-facilitated play (AFP) the experimenter suggested the plot and was involved in the control of the play process while in free play (FP) groups children were to develop the plot and the play process for themselves. We hypothesized that the observed differences would be associated with indicators directly related to initiative and individuality manifestations: unscripted character actions and discussing the development of the play with playmates.

A Kruskal-Wallis test showed that children in free play groups more frequently than children in adult-facilitated play groups exhibit unscripted character actions [χ^2^(1, *N* = 42) = 4.63, *p* = 0.031, η^2^ = 0.113], and speech in the play context [χ^2^(1, *N* = 42) = 6.89, *p* = 0.009, η^2^ = 0.168], and on tendency level more often regulate the behavior of other children [χ^2^(1, *N* = 42) = 3.84, *p* = 0.05, η^2^ = 0.0937]. A higher number of indeterminate behavioral manifestations in free play [χ^2^(1, *N* = 42) = 6.76, *p* = 0.009, η^2^ = 0.164] reflects challenges in coding (observation and interpretation) in this type of paly situation: in the absence of adult guidance, the plot is usually less structured, the play idea can change spontaneously many times, and play interactions are less detailed. No other significant differences were detected.

## Discussion

In our study, we evaluated the reliability and validity of a new tool to analyze the role-play in early childhood, the Play Matrix. The Play Matrix was developed to help researchers to capture the play situation from the child’s perspective using a theory-based framework (Elkonin, 2005; Veresov and Veraksa, 2024; Vygotsky, 2005, 2016) and to provide a mechanism for identifying the effortful control zone of proximal development in play. Unlike other tools for evaluating a collective pretend play, the Play Matrix does not focus on the level of group play, but on a detailed fixation of the particular child play behavior. The analysis of the quantity and quality of play actions, emotional and speech manifestations of the child give evidence for the conclusions about the degree of the child’s involvement in the play process. As demonstrated in the *blinded study*, the Play Matrix reveals a child’s personal experience (what Vygotsky termed “perezhivanie”) and illustrates individual trajectories of executive function development during play. The findings from this study underscore the utility of the Play Matrix as a robust tool for assessing various dimensions of children’s play behavior.

The Play Matrix demonstrated high inter-rater reliability across most indicators, suggesting that it is a consistent and reliable measure for observing and coding play behaviors. The inter-rater reliability turned out to be low for the «Speech in a play» context indicator, which indicates the need for additional clarifications of its definition. It was also not possible to assess the inter-rater reliability for the «Discussing the development of the play with playmates» indicator, as none of the 10 observations recorded this manifestation. This is probably due to the fact that the video recordings of the play sessions did not include a preliminary stage of preparation for the play, as well as the low level of play skills of the observed children, which was noted by all coders.

The analysis revealed significant differences in play behaviors between children with high and low levels of self-regulation. Children with lower self-regulation exhibited more impulsive actions; expressive gestures, and emotional responses; and involvement in emotionally rich group actions. These findings align with existing literature, which suggests that lower self-regulation is associated with higher impulsivity and emotional reactivity (Blair and Razza, 2007; Yogman et al., 2018). The study also highlighted differences in play behaviors across different play situations. Children in free play groups compared to those in adult-facilitated play exhibited more unscripted character actions, engaged in more speech in the play context and on tendency level more often rregulated the behavior of other children These findings support the hypothesis that free play provides greater challenge for children’s involvement in play.

The Play Matrix has proven to be a sensitive and reliable measure for assessing children’s play behaviors and manifestations of effortful control in play. Its comprehensive set of indicators allows for a detailed analysis of play, providing valuable insights into the developmental processes underlying children’s play activities. Although the tool aims to analyze play from the child’s perspective, the Play Matrix relies on adult observation and interpretation of visible behaviors. Complementary qualitative methods, such as brief child interviews, participatory video commentary, or expressive tasks, could strengthen the tool. Continued refinement and validation of the Play Matrix will enhance its utility as a research and educational tool, contributing to the advancement of knowledge in the field of early childhood development. Despite the promising results, the study has several limitations. The sample size was relatively small, which may limit the generalizability of the findings. Due to the small sample size children were divided into two groups – low and high EF – based on K-means clustering, despite the common three-level categorization (low, medium, high) and the risk to overlook the developmental continuum of executive functions. Additionally, technical difficulties resulted in incomplete evaluations by one of the coders, which could have impacted the reliability analysis. Future research should aim to replicate these findings with larger and more diverse samples to further validate the Play Matrix. Moreover, exploring the tool’s applicability in different cultural contexts could provide a broader understanding of its utility and effectiveness. Another promising direction is to adapt the tool to observe children under age three.

## Data Availability

The raw data supporting the conclusions of this article will be made available by the authors, without undue reservation.
